# Case report: Pleasure gone wrong”: a case of endoscopic removal of an extra-long urethral electric cable self-inserted for sexual gratification

**DOI:** 10.3389/fruro.2026.1768406

**Published:** 2026-04-23

**Authors:** Jaisukh Kalathia, Bharti Talreja

**Affiliations:** Deapartment of Urology, Fortune Urology Clinic, Botad, India

**Keywords:** bladder, cystoscopy, electric cable, foreign body, longest

## Abstract

Foreign bodies in lower urinary tract, once regarded as rare, are now increasingly recognized and demand urgent urological intervention. Spectrum of self-inserted urethral objects is remarkably wide, ranging from household items to sharp instruments and cables, with motivations spanning sexual-gratification, autoerotic experimentation and psychiatric illnesses. Clinical presentation varies from acute pain, retention, or haematuria to chronic recurrent infections and unexplained voiding symptoms. We present a case of a 49-year-old male who self-inserted an electric cable, which was successfully retrieved intact through endoscopic intervention within six hours of insertion. This case highlights early recognition, minimally invasive retrieval, and attention to underlying behavioural and psychological risk factors to prevent recurrence.

## Introduction

Foreign bodies in the lower urinary tract, once considered uncommon, are increasingly reported in the literature and represent a urological emergency requiring prompt intervention. Reported urethral insertional objects range widely—from electrical wires, thermometers, nail clippers, needles, hooks, vegetables, bones, and animal parts to sharp items, cable- or tube-like materials, fluids such as glue or hot wax, and even powders like cocaine. Motivations for self-instrumentation of the urethra range from sexual gratification and autoerotic curiosity to psychiatric illness, intoxication, religious or therapeutic beliefs, neurocognitive decline, accidental causes, and even misconceptions about contraception. Acute presentations may include pain, dysuria, urinary retention, or haematuria, while chronic symptoms such as recurrent urinary tract infections (UTI), voiding difficulties, urinary incontinence, pelvic pain, or unexplained haematuria should prompt suspicion of a urethral or bladder foreign body. Although multiple cases of self-inserted urethral objects have been described in the literature, we report a rare case of a 49-year-old male who self-inserted an electric cable; it was successfully retrieved intact via endoscopic intervention and, to our knowledge, represents one of the longest reported in the literature.

## Case report

A 49-year-old male presented to the urology department with complaints of acute-onset voiding difficulty, irritative lower urinary tract symptoms, burning micturition, and lower abdominal pain for the past six hours. Physical examination revealed mild suprapubic tenderness and a blood-tinged urethral meatus on local inspection. On detailed questioning, the patient disclosed a history of self-insertion of foreign objects into the urethra for sexual gratification. He reported that he had been divorced three months earlier and had since been sexually active with escalating autostimulatory practices. Initially, urethral insertion caused discomfort, but over time the sensation transitioned to pleasure, leading him to experiment with various objects.

On the morning of presentation, he inserted an electric cable in an attempt to achieve heightened stimulation. He experienced pleasurable sensations and continued to advance the cable until, unintentionally, the entire length migrated into the urethra, and he could no longer grasp the external end. Multiple self-attempts at retrieval were unsuccessful, after which he developed increasing lower abdominal pain and haematuria. Due to fear and social embarrassment, he initially refrained from informing family members; however, worsening symptoms ultimately prompted him to seek medical attention.

**Figure 1:**
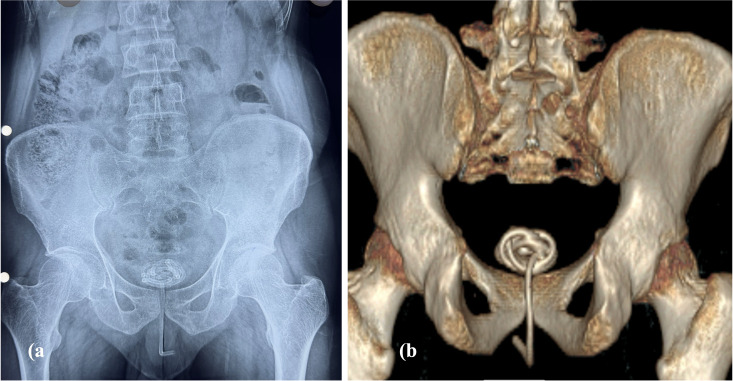
**(A)** X-ray KUB demonstrating a coiled wire superior to pubic symphysis, with one end extending into the urethra **(B)** 3D reconstructed image of CT scan showing coiled wire in bladder.

Ultrasonography revealed a coiled hyperechoic structure consistent with a foreign body within the urinary bladder. A plain X-ray of the kidney–ureter–bladder (KUB) region further demonstrated a coiled wire in the lower abdomen superior to the pubic symphysis, with one end located in the bladder and the other extending into the urethra. (**Figure 1A**) A non-contrast CT scan was obtained to exclude perforation and potential injury to adjacent organs associated with the electric cable. (**Figure 1B**) Routine laboratory investigations, including serum biochemistry, were within normal limits except for a mildly elevated total leukocyte count.

**Figure 2:**
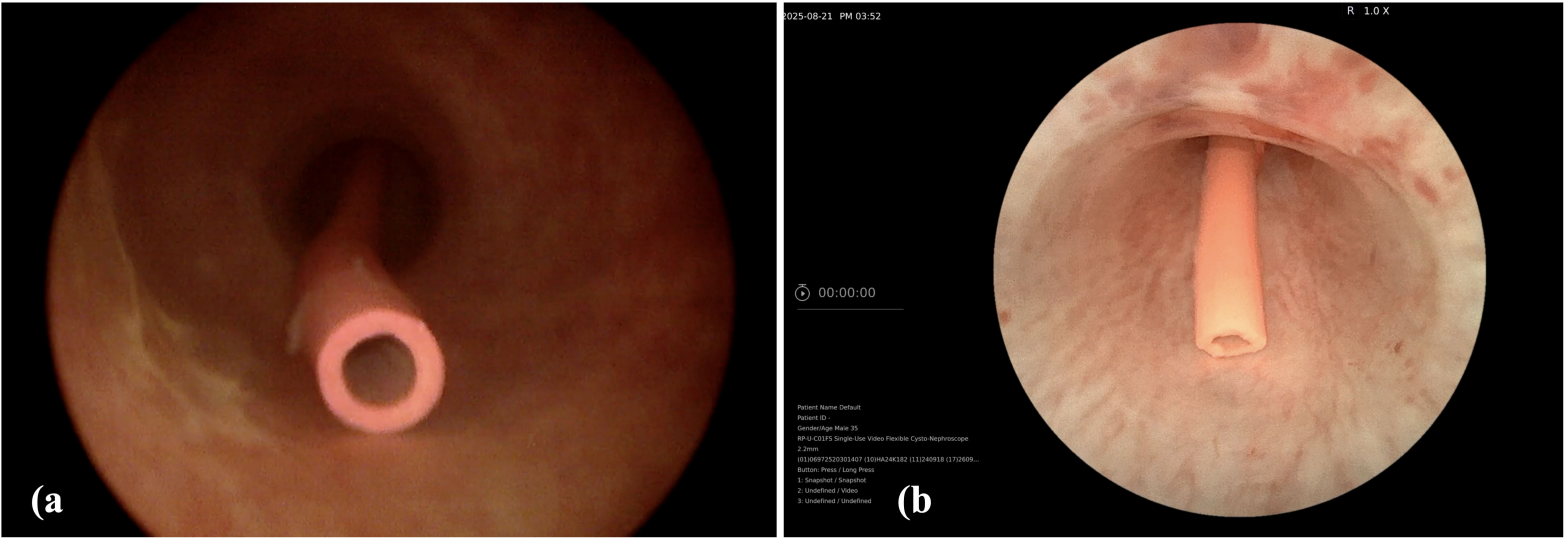
**(A)** Standard rigid cystoscopy **(B)** High-definition digital disposable flexible cystoscopy.

**Figure 3:**
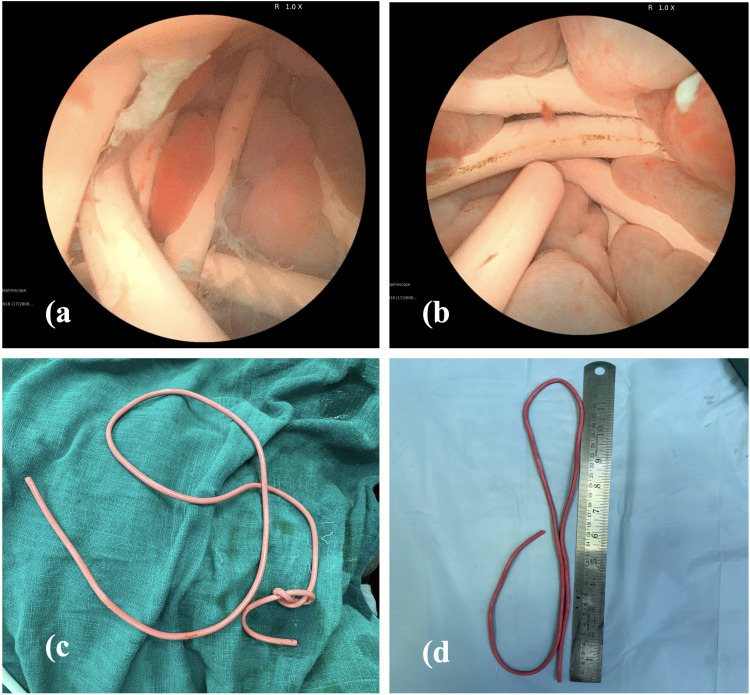
**(A)** Multiple coils of electric cable in bladder **(B)** Electric cable compressing bladder mucosa **(C)** Knotted proximal end of the electric cable **(D)** Uncoiled electric cable measured on scale.

The patient was counselled for office-based flexible diagnostic cystoscopy, which subsequently confirmed the presence of an electric cable consisting only of its only outer sheath, with one end located within the urethra. A gentle attempt at endoscopic retrieval using a grasper was made; however, the patient experienced severe, sharp pain, and further attempts were abandoned. Under general anaesthesia with airway control via intubation and strict aseptic precautions, an initial standard rigid cystoscopy was performed in lithotomy position, revealing the outer sheath of an electric cable lodged within the urethra (**Figure 2A**). For clearer identification and characterization of the foreign body, a high-definition (HD) digital disposable flexible cystoscopy (Redpine^®^) was subsequently undertaken. This provided superior delineation of the pink-coloured electric cable, confirming that one end of the foreign body was positioned within the urethra (**Figure 2B**). Advancing the flexible cystoscope into the bladder demonstrated multiple coils of the same pink-coloured cable completely filling the bladder cavity (**Figure 3A**), causing marked compression of the bladder mucosa and hindering adequate bladder distension. (**Figure 3B**) A flexible grasper was then introduced through the working channel of the HD digital disposable flexible cystoscope to facilitate therapeutic manoeuvres aimed at securely grasping and retrieving the cable.

An initial attempt to grasp and extract the distal end of the cable with forceps successfully delivered the tip to the urethral meatus; however, the remainder of the cable could not be withdrawn gently due to resistance within the urethra. Additional xylocaine jelly was instilled alongside the cable, and a firm, controlled traction was applied, resulting in complete extraction of the foreign body. Notably, the proximal end of the cable was knotted, accounting for the resistance encountered during removal. (**Figure 3C**). The uncoiled cable measured 75 cm in length. (**Figure 3D**). Repeat cystoscopy was performed to assess for urethral or bladder trauma and to exclude retained fragments, with no residual foreign material identified.

The postoperative course was uneventful. The Foley catheter was removed on day one, with satisfactory voiding thereafter. The patient was discharged in stable condition on a 10-day course of prophylactic antibiotics and referred for psychiatric evaluation, including psychosexual assessment, with initiation of appropriate antidepressant therapy.

## Discussion

Self-insertion of foreign bodies into the male urethra is more common than previously believed, with numerous cases routinely reported in the literature worldwide. These presentations often pose significant challenges in endoscopic retrieval and management. Over the years, a remarkably wide spectrum of inserted objects has been documented, including electrical wires, thermometers, nail clippers, needles, hooks, vegetables, bones, animal parts, sharp instruments, cable- or tube-like materials, as well as fluids such as glue or hot wax, and even powders like cocaine (1). The motivations for self-instrumentation vary widely; however, reported cases are commonly associated with underlying psychiatric disorders, cognitive impairment, intoxication, sexual experimentation, or autoerotic stimulation (2).

Clinical presentation may vary widely. Acute symptoms often include pain, dysuria, urinary retention, or haematuria, whereas chronic presentations may involve recurrent UTIs, voiding difficulties, urinary incontinence, pelvic discomfort, or unexplained haematuria. Nevertheless, some patients remain asymptomatic or present with non-specific complaints, and many are reluctant to disclose an accurate history, further complicating diagnosis.

Diagnosis is based on a meticulous clinical history and focused physical examination. Imaging modalities such as KUB radiography and ultrasonography play an important role in confirming the presence and location of foreign bodies (3). Office-based flexible cystoscopy is particularly valuable, as it permits direct visualization of the urethra and bladder, enables precise characterization of the foreign object, and in many instances allows simultaneous therapeutic intervention (4). Recent advances in high-definition disposable flexible cystoscopy now provide exceptionally sharp imagery with accurate colour reproduction, resulting in near–picture-perfect visualization and detailed delineation of the entire urethra. This enhanced clarity facilitates the close identification of foreign bodies and any associated mucosal injury. In our case, these advantages were well demonstrated using a disposable (Redpine^®^) flexible HD cystoscope.

Foreign body removal should be tailored to the size, morphology, and location of the object, with a clear emphasis on minimizing urethral and bladder trauma. In most instances, transurethral extraction with cystoscopic grasping instruments is sufficient. Larger or firmly impacted objects may require a suprapubic approach. Endoscopic retrieval remains the gold standard for intraluminal genitourinary foreign bodies owing to its minimally invasive profile, high success rate, and low complication burden. Flexible or rigid cystoscopy allows direct visualization and often facilitates simultaneous therapeutic intervention. Open or combined surgical techniques are reserved for cases where endoscopic measures fail or when complications—such as perforation, migration, or significant local tissue injury—necessitate escalation. Preservation of urethral integrity is critical, as iatrogenic trauma increases the risk of strictures, infection, and long-term morbidity. In our case, endoscopic extraction was completed successfully without causing any injury to the urethra or bladder.

The patient was referred for comprehensive psychological evaluation to exclude underlying psychiatric disorders associated with excessive sexual gratification and to mitigate the risk of future sexual experimentation or autoerotic practices that may result in self-injury.

## Conclusion

Self-inserted urethral foreign bodies present a complex clinical challenge that requires timely diagnosis, careful endoscopic management, and a strong emphasis on preventing iatrogenic injury. Endoscopic retrieval remains the preferred modality in the majority of cases, offering high success rates with minimal morbidity when performed with meticulous technique. Long-term outcomes are optimized not only by atraumatic removal but also by addressing underlying psychological or behavioural drivers to reduce the risk of recurrence.

## Data Availability

The original contributions presented in the study are included in the article/supplementary material. Further inquiries can be directed to the corresponding author.
